# Supplementary figures and images for: Clinical and biological clusters of sepsis patients using hierarchical clustering
Source: PLoS One. 2021 Aug 4;16(8):e0252793. doi: 10.1371/journal.pone.0252793 (PMC8336799; doi:10.1371/journal.pone.0252793)

S1 Fig : Flow Chart.


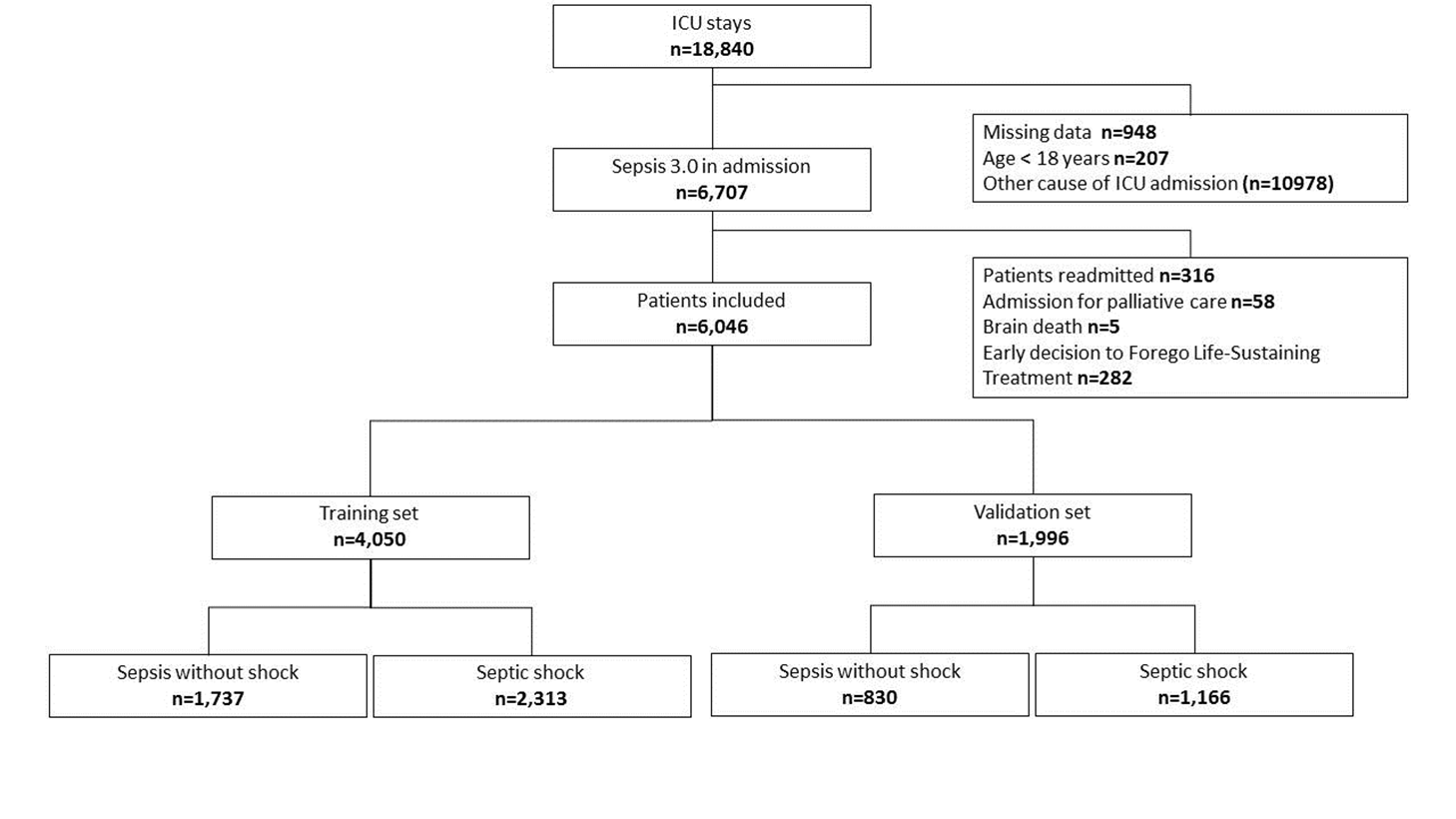

Supplement: S1 Fig — (DOCX) [file pone.0252793.s001.docx]
