# Supplementary material for: Clinical and biological clusters of sepsis patients using hierarchical clustering
Source: PLoS One. 2021 Aug 4;16(8):e0252793. doi: 10.1371/journal.pone.0252793 (PMC8336799; doi:10.1371/journal.pone.0252793)
Supplement: S2 Fig — Dendrogram obtained after application of hierarchical clustering analysis by accounting for the 51 dimensions of the multiple correspondence analysis. The vertical axis of the dendrogram represents the distance between clusters. The horizontal vertical axis represents the patients and clusters. Each junction between two clusters is represented on the graph by the split of a vertical line into two vertical lines. The vertical position of the split, shown by the short horizontal bar, gives the distance between the two clusters. The red line shows the cut level that determines the number of clusters. The indices used to determine this cut level, Semi partial R-Squared, the Squared-R, the Pseudo F statistic and the Pseudo t2 statistic, are presented in S3 Fig. (DOCX) [file pone.0252793.s002.docx]

S2 Fig : Dendrogram of ascending hierarchical clustering analysis (performed in training set).


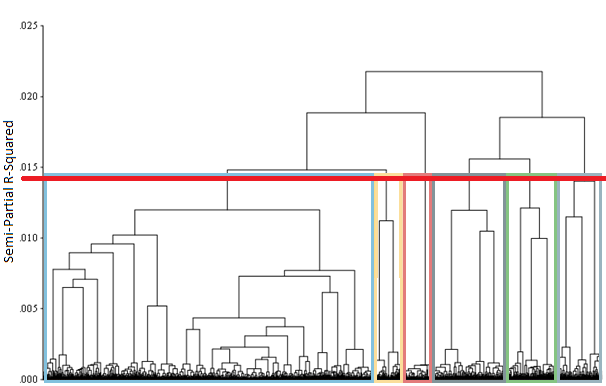


Dendrogram obtained after application of hierarchical clustering analysis by accounting for the 51 dimensions of the multiple correspondence analysis. The vertical axis of the dendrogram represents the distance between clusters. The horizontal vertical axis represents the patients and clusters. Each junction between two clusters is represented on the graph by the split of a vertical line into two vertical lines. The vertical position of the split, shown by the short horizontal bar, gives the distance between the two clusters. The red line shows the cut level that determines the number of clusters. The indices used to determine this cut level, Semi partial R-Squared, the Squared-R, the Pseudo F statistic and the Pseudo t2 statistic, are presented in S3 Fig.
