# Supplementary material for: Clinical and biological clusters of sepsis patients using hierarchical clustering
Source: PLoS One. 2021 Aug 4;16(8):e0252793. doi: 10.1371/journal.pone.0252793 (PMC8336799; doi:10.1371/journal.pone.0252793)
Supplement: S3 Fig — A: The Semi partial R-Squared given the decrease in the proportion of variance accounted of resulting from joining the two clusters. B: The Squared-R is the proportion of variance accounted for by the cluster. C: The Pseudo F statistic measuring the separation among all clusters at the current level. D: The Pseudo t2 statistic measuring the separation between the two clusters most recently joined. There is no consensus in the literature on the final choice of the number of clusters regardless of the clustering method. According to the different criteria (Semi partial R-Squared = 4 to 6 clusters, R squared = upper 10, Pseudo F statistic = 4 to 7 clusters, Pseudo t2 statistic = 2, 4 or 6 clusters), the number of six clusters is the best trade-off between goodness of fit criteria and clinical interpretation. (DOCX) [file pone.0252793.s003.docx]

S3 Fig : Reprentation of indices used to determine the dendrogram cut level (performed in training set).


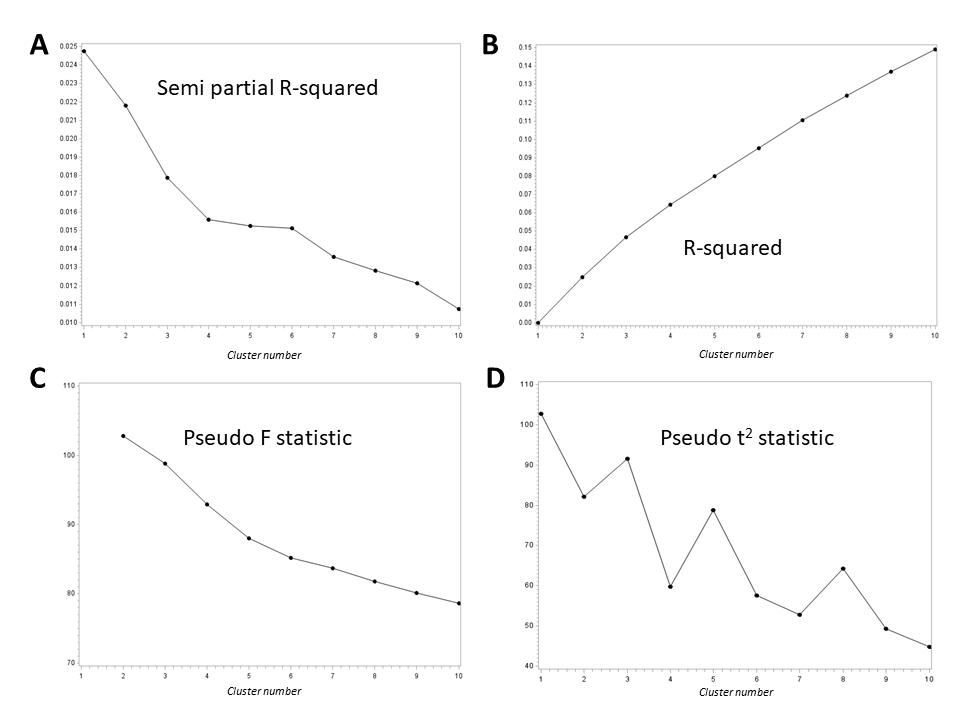
**A**: The Semi partial R-Squared given the decrease in the proportion of variance accounted of resulting from joining the two clusters. **B**: The Squared-R is the proportion of variance accounted for by the cluster. **C**: The Pseudo F statistic measuring the separation among all clusters at the current level. **D**: The Pseudo t2 statistic measuring the separation between the two clusters most recently joined.

There is no consensus in the literature on the final choice of the number of clusters regardless of the clustering method. According to the different criteria (Semi partial R-Squared = 4 to 6 clusters, R squared = upper 10, Pseudo F statistic = 4 to 7 clusters, Pseudo t^2^ statistic = 2, 4 or 6 clusters), the number of six clusters is the best trade-off between goodness of fit criteria and clinical interpretation.
