# Supplementary material for: Clinical and biological clusters of sepsis patients using hierarchical clustering
Source: PLoS One. 2021 Aug 4;16(8):e0252793. doi: 10.1371/journal.pone.0252793 (PMC8336799; doi:10.1371/journal.pone.0252793)
Supplement: S5 Fig — Definition of abbreviations: MCA: Multiple correspondence analysis; HC: Hierarchical clustering; Each patient was represented using his individual coordinates in these dimensions. Patient’s assignment to a cluster was obtained after HC application. A: Axes correspond to the first and second dimension of MCA. Each patient was represented using his individual coordinates in these dimensions. B: Axes correspond to the third and fourth dimension of MCA. (DOCX) [file pone.0252793.s005.docx]

S5 Fig: Point cloud of patients representing the 6 clusters in the first four dimensions of the MCA (performed in training set).


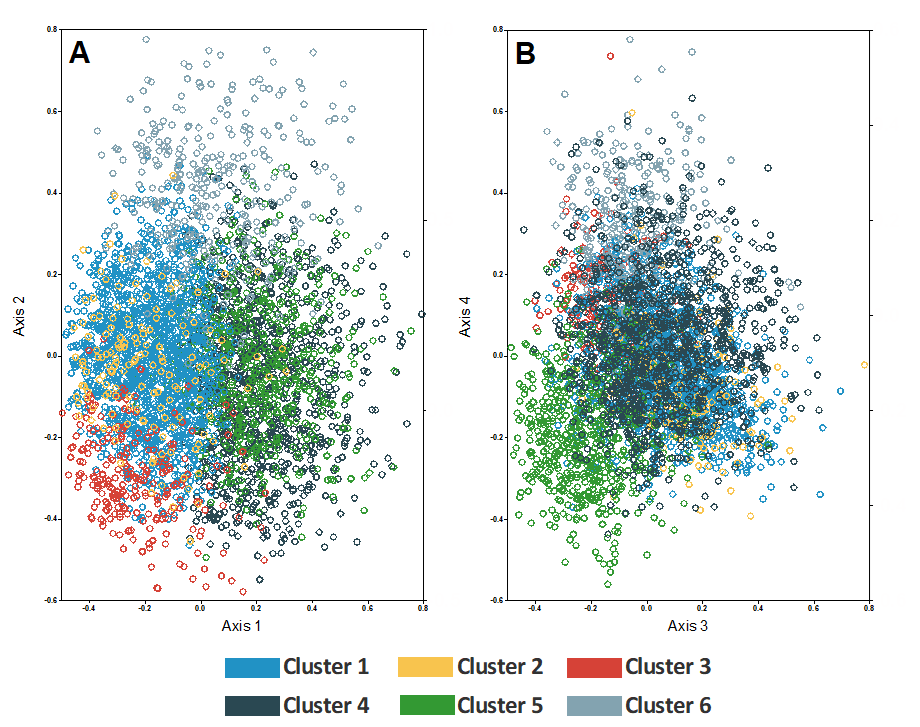


Definition of abbreviations: MCA: multiple correspondence analysis; HC: hierarchical clustering;

Each patient was represented using his individual coordinates in these dimensions. Contribution of each variable to the first four dimensions of the MCA is available in Figure 1. Patient’s assignment to a cluster was obtained after HC application. **A:** Axes correspond to the first and second dimension of MCA. Each patient was represented using his individual coordinates in these dimensions. **B:** Axes correspond to the third and fourth dimension of MCA.
