# Supplementary material for: Clinical and biological clusters of sepsis patients using hierarchical clustering
Source: PLoS One. 2021 Aug 4;16(8):e0252793. doi: 10.1371/journal.pone.0252793 (PMC8336799; doi:10.1371/journal.pone.0252793)
Supplement: S6 Fig — A: Figure of cluster 1; B: Figure of cluster 2; C: Figure of cluster 3; D: Figure of cluster 4; E: Figure of cluster 5; F: Figure of cluster 6; Definition of abbreviations: COPD = chronic obstructive pulmonary disease; HIV = human immunodeficiency virus; AIDS = acquired immune deficiency syndrome; Leucopenia was defined by a leukocyte count below 500/mm3; Hyperlactatemia was defined by a blood lactate concentration greater than 2 mmol/l; Median of SOFA score by organ was used to define organ dysfunction in binary variable. Associations with leucopenia and bronchial could not be calculated due to insufficient numbers in this cluster. (DOCX) [file pone.0252793.s006.docx]

S6 Fig: Probability for variables for belonging to one cluster assessed using univariable logistic regression (performed in training set).

**A**: figure of cluster 1 ; **B**: figure of cluster 2; **C**: figure of cluster 3 ; **D**: figure of cluster 4; **E**: figure of cluster 5 ; **F**: figure of cluster 6; *Definition of abbreviations*: COPD = chronic obstructive pulmonary disease; HIV = human immunodeficiency virus; AIDS = acquired immune deficiency syndrome; Leucopenia was defined by a leukocyte count below 500/mm3; Hyperlactatemia was defined by a blood lactate concentration greater than 2 mmol/l; Median of SOFA score by organ was used to define organ dysfunction in binary variable. Associations with leucopenia and bronchial could not be calculated due to insufficient numbers in this cluster.
