# Supplementary material for: Clinical and biological clusters of sepsis patients using hierarchical clustering
Source: PLoS One. 2021 Aug 4;16(8):e0252793. doi: 10.1371/journal.pone.0252793 (PMC8336799; doi:10.1371/journal.pone.0252793)
Supplement: S7 Fig — Definition of abbreviations: OR: Odds ratio; The analysis was performed after exclusion of patients without septic shock. A logistic regression was used to determine the odds ratio; Data are reported as odds ratios ± 95% confidence intervals, presented from lowest to highest; Cluster 3 was used as reference class; Adjusted mortality were adjusted using SOFA score at admission and year of ICU admission. Cluster 1 = young patients without any comorbidities, admitted in ICU for community-acquired pneumonia; Cluster 2 = young patients without any comorbidities, admitted in ICU for meningitis or encephalitis; Cluster3 = elderly patients with COPD, admitted in ICU for bronchial infection with few organ failures; Cluster 4 = elderly patients with several comorbidities and organ failures; Cluster 5 = patients admitted after surgery with a nosocomial infection; Cluster 6 = young patients with immunosuppressive disease or therapy, such as AIDS, chronic steroid therapy or hematological malignancy. (DOCX) [file pone.0252793.s007.docx]

S7 Fig : Risks of mortality at 28 days, 90 days and one-year according to the cluster assignment in patients with septic shock (performed in training set).

*Definition of abbreviations:* HR: hazard ratio; The analysis was performed after exclusion of patients without septic shock. A Cox model was used to determine the hazard ratio; Data are reported as HR ± 95% confidence intervals, presented from lowest to highest; Cluster 3 was used as reference class; Adjusted mortality were adjusted using SOFA score at admission and year of ICU admission. **Cluster 1 =** young patients without any comorbidities, admitted in ICU for community-acquired pneumonia; **Cluster 2 =** young patients without any comorbidities, admitted in ICU for meningitis or encephalitis; **Cluster3 =** elderly patients with COPD, admitted in ICU for bronchial infection with few organ failures; **Cluster 4** = elderly patients with several comorbidities and organ failures; **Cluster 5 =** patients admitted after surgery with a nosocomial infection; **Cluster 6** = young patients with immunosuppressive disease or therapy, such as AIDS, chronic steroid therapy or hematological malignancy.
