# Supplementary material for: Clinical and biological clusters of sepsis patients using hierarchical clustering
Source: PLoS One. 2021 Aug 4;16(8):e0252793. doi: 10.1371/journal.pone.0252793 (PMC8336799; doi:10.1371/journal.pone.0252793)
Supplement: S8 Fig — Definition of abbreviations: ICU = intensive care unit; The binary tree was built in the training set using Breiman methods [22] with Rpart package version 4.1–10 [23], R version 3.1.0. The structure is similar to a real tree, from the bottom up: There is a root, where the first split happens. After each split, two new nodes are created. Each node contains only a subset of the patients with a minimum size of 20. The partitions of the data, which are not split any more, are called terminal nodes or leafs. The second stage of the procedure consists to prune the tree using cross-validation. Pruning means to shorten the tree, which makes trees more compact and avoids over fitting to the training data. Each split is examined, if it brings a reliable improvement. The six variables used by the binary tree are: Lung infection, Surgical admission, Hematological malignancy, Bronchial infection, Chronic heart failure and Meningeal infection. The accuracy of the binary tree evaluated in the training dataset was available on S3 Table. Cluster 1 = young patients without any comorbidities, admitted in ICU for community-acquired pneumonia; Cluster 2 = young patients without any comorbidities, admitted in ICU for meningitis or encephalitis; Cluster3 = elderly patients with COPD, admitted in ICU for bronchial infection with few organ failures; Cluster 4 = elderly patients with several comorbidities and organ failures; Cluster 5 = patients admitted after surgery with a nosocomial infection; Cluster 6 = young patients with immunosuppressive disease or therapy, such as AIDS, chronic steroid therapy or hematological malignancy. (DOCX) [file pone.0252793.s008.docx]

S8 Fig: Binary tree using variables available at the ICU admission.


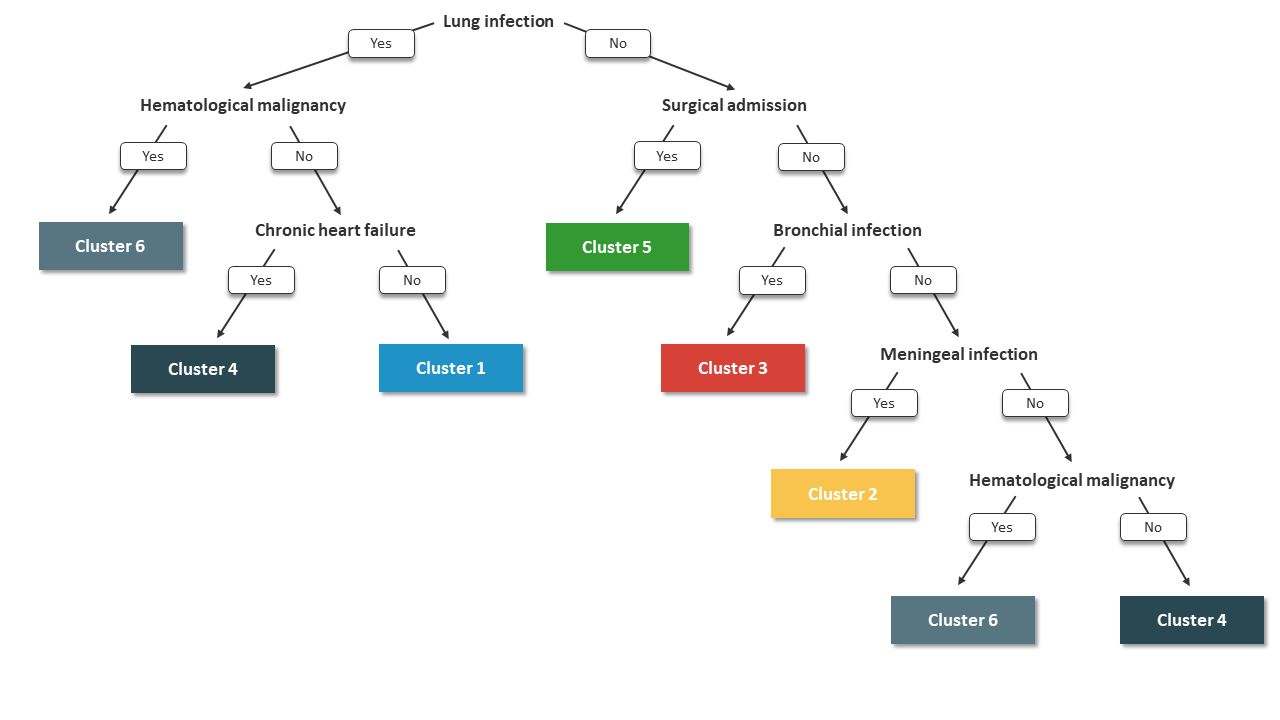


*Definition of abbreviations:* ICU = intensive care unit; The binary tree was built in the training set using Breiman methods [22] with Rpart package version 4.1-10 [23], R version 3.1.0. The structure is similar to a real tree, from the bottom up: there is a root, where the first split happens. After each split, two new nodes are created. Each node contains only a subset of the patients with a minimum size of 20. The partitions of the data, which are not split any more, are called terminal nodes or leafs. The second stage of the procedure consists to prune the tree using cross-validation. Pruning means to shorten the tree, which makes trees more compact and avoids over fitting to the training data. Each split is examined, if it brings a reliable improvement. The six variables used by the binary tree are : Lung infection, Surgical admission, Hematological malignancy, Bronchial infection, Chronic heart failure and Meningeal infection. The accuracy of the binary tree evaluated in the training dataset was available on S3 Table. Cluster 1 = young patients without any comorbidities, admitted in ICU for community-acquired pneumonia; Cluster 2 = young patients without any comorbidities, admitted in ICU for meningitis or encephalitis; Cluster3 = elderly patients with COPD, admitted in ICU for bronchial infection with few organ failures; Cluster 4 = elderly patients with several comorbidities and organ failures; Cluster 5 = patients admitted after surgery with a nosocomial infection; Cluster 6 = young patients with immunosuppressive disease or therapy, such as AIDS, chronic steroid therapy or hematological malignancy.
