# Supplementary material for: Clinical and biological clusters of sepsis patients using hierarchical clustering
Source: PLoS One. 2021 Aug 4;16(8):e0252793. doi: 10.1371/journal.pone.0252793 (PMC8336799; doi:10.1371/journal.pone.0252793)
Supplement: S10 Fig — Definition of abbreviations: MCA = multiple correspondence analysis; COPD = chronic obstructive pulmonary disease; ICU = intensive care unit; SOFA = Sequential Organ Failure Assessment score; The contribution is expressed as a percentage. The representation is limited to the first 20 variables that contribute most to the dimension of the 63 used. The variables represented are classified in descending order of contribution. (DOCX) [file pone.0252793.s010.docx]

S10 Fig : Contribution of each variable to the first four dimensions of the MCA (performed in validation set).

*Definition of abbreviations:* MCA= multiple correspondence analysis; COPD = chronic obstructive pulmonary disease; ICU= intensive care unit; SOFA= Sequential Organ Failure Assessment score; The contribution is expressed as a percentage. The representation is limited to the first 20 variables that contribute most to the dimension of the 63 used. The variables represented are classified in descending order of contribution.
