# Supplementary material for: Clinical and biological clusters of sepsis patients using hierarchical clustering
Source: PLoS One. 2021 Aug 4;16(8):e0252793. doi: 10.1371/journal.pone.0252793 (PMC8336799; doi:10.1371/journal.pone.0252793)
Supplement: S1 Table — Definition of abbreviations: COPD = chronic obstructive pulmonary disease; HIV = human immunodeficiency virus; AIDS = acquired immune deficiency syndrome; NF GNB: Non-fermentative Gram negative bacilli; MDRO: Multi-drug resistance organisms (refer to vancomycin-resistant enterococci, methicillin-resistant Staphylococcus aureus, extended-spectrum β-lactamase-producing Enterobacteriaceae, AmpC-producing Enterobacteriaceae, Pseudomonas aeruginosa resistant to more than two antimicrobial families, Stenotrophomonas maltophilia); Values in Numbers (%) or median [IQR]. (DOCX) [file pone.0252793.s011.docx]

S1 Table: The 63 variables included in the cluster analysis, distribution and missing values.

| **Variable** | **All dataset**  **n=6.046** | **Missing** | **Training set**  **n=4.050** | **Validation set**  **n=1.996** |
| --- | --- | --- | --- | --- |
| ***Host characteristics*** |  |  |  |  |
| Age (years) | 65 [52-76] | 0 (0%) | 65 [52-76] | 65 [53-76] |
| Sex (Male) | 3763 (62%) | 0 (0%) | 2491 (62%) | 1272 (64%) |
| Weight (kg) | 70 [60-81] | 696 (11%) | 70 [60-82] | 70 [60-82] |
| Malnutrition | 328 (5%) | 0 (0%) | 230 (6%) | 98 (5%) |
| Alcohol abuse | 931 (15%) | 0 (0%) | 625 (15%) | 306 (15%) |
| Not complicated diabetes | 699 (12%) | 0 (0%) | 491 (12%) | 208 (10%) |
| Complicated diabetes | 301 (5%) | 0 (0%) | 213 (5%) | 88 (4%) |
| Chronic heart failure | 1105 (18%) | 0 (0%) | 749 (18%) | 356 (18%) |
| Chronic kidney disease | 477 (8%) | 0 (0%) | 315 (8%) | 162 (8%) |
| Liver cirrhosis | 510 (8%) | 0 (0%) | 352 (9%) | 158 (8%) |
| COPD | 1354 (22%) | 0 (0%) | 892 (22%) | 462 (23%) |
| Hematological malignancy | 571 (9%) | 0 (0%) | 387 (10%) | 184 (9%) |
| HIV/AIDS or Transplant | 344 (6%) | 0 (0%) | 231 (6%) | 113 (6%) |
| Solid tumor | 773 (13%) | 0 (0%) | 520 (13%) | 253 (13%) |
| Chronic steroid therapy | 363 (6%) | 0 (0%) | 250 (6%) | 113 (6%) |
| Charlson score | 3 [2-4] | 0 (0%) | 3 [2-4] | 3 [2-4] |
| ***ICU Admission*** |  | 0 (0%) |  |  |
| Medical admission | 4848 (80%) |  | 3239 (80%) | 1609 (81%) |
| Unscheduled surgery | 943 (16%) |  | 636 (16%) | 307 (15%) |
| Scheduled surgery | 255 (4%) |  | 175 (4%) | 80 (4%) |
| ***Source of infection*** |  |  |  |  |
| Pulmonary | 3118 (52%) | 0 (0%) | 2073 (51%) | 1045 (52%) |
| Bronchial | 362 (6%) | 0 (0%) | 240 (6%) | 122 (6%) |
| Urinary tract | 642 (11%) | 0 (0%) | 427 (11%) | 215 (11%) |
| Surgical abdomen | 650 (11%) | 0 (0%) | 447 (11%) | 203 (10%) |
| Medical abdomen | 238 (4%) | 0 (0%) | 148 (4%) | 90 (5%) |
| Soft tissues | 247 (4%) | 0 (0%) | 172 (4%) | 75 (4%) |
| Meningeal encephalitis | 232 (4%) | 0 (0%) | 150 (4%) | 82 (4%) |
| Miscellaneous sites | 325 (5%) | 0 (0%) | 217 (5%) | 108 (5%) |
| Unknown site | 386 (6%) | 0 (0%) | 275 (7%) | 111 (6%) |
| ***Infection micro-organisms*** |  |  |  |  |
| *Escherichia coli* | 894 (15%) | 0 (0%) | 597 (15%) | 297 (15%) |
| Other *Enterobacteriaceae* | 620 (10%) | 0 (0%) | 413 (10%) | 207 (10%) |
| *Pseudomonas* spp. and other NF GNB | 409 (7%) | 0 (0%) | 290 (7%) | 119 (6%) |
| *Streptococcus pneumoniae* | 510 (8%) | 0 (0%) | 351 (9%) | 159 (8%) |
| *Enterococcus* and *Streptococcus* | 588 (10%) | 0 (0%) | 389 (10%) | 199 (10%) |
| *Staphylococcus aureus* | 521 (9%) | 0 (0%) | 371 (9%) | 150 (8%) |
| Fungus | 152 (3%) | 0 (0%) | 100 (2%) | 52 (3%) |
| Virus | 160 (3%) | 0 (0%) | 114 (3%) | 46 (2%) |
| Other pathogens | 1092 (18%) | 0 (0%) | 556 (14%) | 354 (18%) |
| Unknown pathogen | 2373 (39%) | 0 (0%) | 1557 (38%) | 816 (41%) |
| Bacteriemia | 980 (16%) | 0 (0%) | 670 (17%) | 308 (15%) |
| Nosocomial | 2034 (34%) | 0 (0%) | 1385 (34%) | 649 (33%) |
| MDRO | 515 (9%) | 0 (0%) | 361 (9%) | 154 (8%) |
| ***Host response*** |  |  |  |  |
| Myocardial dysfunction | 1190 (20%) | 0 (0%) | 785 (19%) | 405 (20%) |
| Cardiac arrest before admission | 308 (5%) | 0 (0%) | 215 (5%) | 93 (5%) |
| Hyperglycemia (>11 mmol/l) | 986 (16%) | 0 (0%) | 679 (17%) | 307 (15%) |
| Hypoglycemia (<3 mmol/l) | 188 (3%) | 0 (0%) | 124 (3%) | 64 (3%) |
| Body temperature (°C) | 38.2 [37.5-39] | 0 (0%) | 38.2 [37.5-39] | 38.2 [37.5-39] |
| New atrial fibrillation | 936 (15%) | 0 (0%) | 645 (16%) | 291 (15%) |
| Recurrent atrial fibrillation | 365 (6%) | 0 (0%) | 233 (6%) | 132 (7%) |
| Heart rate (beats/min) | 116 [100-134] | 96 (2%) | 116 [100-134] | 118 [102-135] |
| Respiratory rate (breaths/min) | 25 [20-32] | 253 (4%) | 25 [20-32] | 25 [20-32] |
| Sodium blood level (mmol/l) | 137 [133-141] | 12 (1%) | 137 [133-141] | 137 [133-141] |
| Potassium blood level (mmol/l) | 4 [3.5-4.6] | 24 (1%) | 4 [3.5-4.6] | 4 [3.5-4.5] |
| Bicarbonate blood level (mmol/l) | 21 [17-25] | 70 (1%) | 21 [17-25] | 21 [17-25] |
| Hematocrit (%) | 33 [28-39] | 453 (7%) | 33 [28-38.6] | 33 [28-38.2] |
| Prothrombin time (%) | 66 [50-80] | 431 (7%) | 66 [50-80] | 66 [51-80] |
| Leukocytes (x10^3^/mm3), | 12.5 [7.5-18.5] | 48 (1%) | 12.0 [7.5-18.5] | 12,5 [7,4-18,8] |
| Fluid replacement >50 ml/kg | 1177 (19%) | 0 (0%) | 753 (19%) | 424 (21%) |
| ***Organ failure*** |  |  |  |  |
| Vasopressor at admission | 3479 (58%) | 0 (0%) | 2313 (57%) | 1166 (58%) |
| Glasgow Coma Score | 15 [9-15] | 0 (0%) | 15 [9-15] | 15 [9-15] |
| Creatinine level (µmol/l) | 104 [73-173] | 0 (0%) | 104 [73-175] | 103 [74-169] |
| Platelets count (x10^3^/mm^3^) | 196 [117-281] | 0 (0%) | 198 [117-284] | 191 [117-276] |
| PaO2/FiO2 ratio (mmHg) | 236 [145-381] | 0 (0%) | 239 [146-388] | 230 [140-369] |
| Bilirubin level (mmol/l) | 13 [8-24] | 0 (0%) | 14 [8-25] | 14 [8-23] |
| Blood lactate level (mmol/l) | 2.0 [1.3-3.5] | 1.113 (18%) | 2.0 [1.3-3.5] | 1.9 [1.3-3.4] |

*Definition of abbreviations:* COPD = chronic obstructive pulmonary disease; HIV = human immunodeficiency virus; AIDS = acquired immune deficiency syndrome; NF GNB: non-fermentative Gram negative bacilli; MDRO: multi-drug resistance organisms (refer to vancomycin-resistant enterococci, methicillin-resistant *Staphylococcus aureus*, extended-spectrum β-lactamase-producing *Enterobacteriaceae*, AmpC-producing *Enterobacteriaceae*, *Pseudomonas aeruginosa* resistant to more than two antimicrobial families, *Stenotrophomonas maltophilia*); Values in Numbers (%) or median [IQR].
