# Supplementary material for: Clinical and biological clusters of sepsis patients using hierarchical clustering
Source: PLoS One. 2021 Aug 4;16(8):e0252793. doi: 10.1371/journal.pone.0252793 (PMC8336799; doi:10.1371/journal.pone.0252793)
Supplement: S2 Table — Definition of abbreviations: ICU = intensive care unit; IQR = interquartile range; Free days were censored at 28 days; A ventilator-free day refers to a day without invasive or non-invasive mechanical ventilation or death; A catecholamine-free day refers to a day without to vasoactive or inotropic agent or death; An organ system failure-free day refers to a day without SOFA score upper zero or death; Cluster 1 = young patients without any comorbidities, admitted in ICU for community-acquired pneumonia; Cluster 2 = young patients without any comorbidities, admitted in ICU for meningitis or encephalitis; Cluster 3 = elderly patients with COPD admitted in ICU for bronchial infection with few organ failures; Cluster 4 = elderly patients, with several comorbidities and organ failures; Cluster 5 = patients admitted after surgery with a nosocomial infection; Cluster 6 = young patients with immunosuppressive disease or therapy, such as AIDS, chronic steroid therapy or hematological malignancy. Values in Numbers (%) or median [IQR]. P- values were obtained by Analysis of variance or Chi-2 test. (DOCX) [file pone.0252793.s012.docx]

S2 Table: Outcomes of patients (performed in training set).

| **Outcomes** | **Training set**  **n=4,050** | **Cluster 1**  **n=1,603** | **Cluster 2**  **n=149** | **Cluster 3**  **n=243** | **Cluster 4**  **n=1,094** | **Cluster 5**  **n=623** | **Cluster 6**  **n=338** | ***P*-Value** |
| --- | --- | --- | --- | --- | --- | --- | --- | --- |
| 28-day mortality | 982 (24%) | 279 (17%) | 27 (18%) | 37 (15%) | 384 (35%) | 150 (24%) | 105 (31%) | <.001 |
| 90-day mortality | 1,267 (31%) | 376 (23%) | 37 (25%) | 42 (18%) | 470 (43%) | 206 (33%) | 135 (40%) | <.001 |
| 1-year mortality | 1,366 (34%) | 406 (25%) | 40 (27%) | 46 (19%) | 503 (46%) | 217 (35%) | 154 (46%) | <.001 |
| ICU stay (days) | 7 [4-15] | 7 [4-15] | 10 [5-16] | 7 [4-12] | 8 [4-15] | 9 [5-18] | 5 [3-12] | <.001 |
| Hospital stay (days) | 22 [12-41] | 21 [11-38] | 23 [15-41] | 19 [12-31] | 21 [11-38] | 31 [17-54] | 26 [12-43] | <.001 |
| Ventilator-free days (days) | 21 [3-28] | 23 [6-28] | 20 [3-28] | 28 [16-28] | 18 [1-28] | 20 [1-26] | 22.5 [3-28] | <.001 |
| Catecholamine-free days (days) | 27 [16-28] | 28 [23-28] | 28 [24-28] | 28 [25-28] | 24 [5-28] | 25 [14-28] | 26 [8-28] | <.001 |
| Renal replacement therapy before 28-day | 1386 (34%) | 364 (23%) | 37 (25%) | 40 (16%) | 557 (51%) | 236 (38%) | 152 (45%) | <.001 |
| Organ system failure-free days (days) | 17 [0-24] | 19 [1-24] | 17 [1-23] | 21 [10-24] | 11 [0-22] | 15 [0-23] | 18 [0-24] | <.001 |

*Definition of abbreviations:* ICU = intensive care unit; IQR = interquartile range; Free days were censored at 28 days; A ventilator-free day refers to a day without invasive or non-invasive mechanical ventilation or death; A catecholamine-free day refers to a day without to vasoactive or inotropic agent or death; An organ system failure-free day refers to a day without SOFA score upper zero or death; **Cluster 1** = young patients without any comorbidities, admitted in ICU for community-acquired pneumonia; **Cluster 2** = young patients without any comorbidities, admitted in ICU for meningitis or encephalitis; **Cluster 3** = elderly patients with COPD admitted in ICU for bronchial infection with few organ failures; **Cluster 4** = elderly patients, with several comorbidities and organ failures; **Cluster 5** = patients admitted after surgery with a nosocomial infection; **Cluster 6** = young patients with immunosuppressive disease or therapy, such as AIDS, chronic steroid therapy or hematological malignancy. Values in Numbers (%) or median [IQR]. *P-* values were obtained by Analysis of variance or Chi-2 test.
