# Supplementary material for: Clinical and biological clusters of sepsis patients using hierarchical clustering
Source: PLoS One. 2021 Aug 4;16(8):e0252793. doi: 10.1371/journal.pone.0252793 (PMC8336799; doi:10.1371/journal.pone.0252793)
Supplement: S3 Table — Definition of abbreviations: Truly assigned = Number of patients correctly assigned as belonging to the cluster according to the total number of patients in the cluster; Falsely assigned = Number of patients incorrectly labeled as belonging to the cluster according to the total number of patients out of the cluster; Se = Sensitivity; Sp = Specificity; AUC = Area under the Receiver Operating Characteristic curve; IC95% = 95% confidence intervals; Cluster 1 = young patients, without any comorbidities, admitted in ICU for community-acquired pneumonia; Cluster 2 = young patients, without any comorbidities, admitted in ICU for meningitis or encephalitis; Cluster 3 = elderly patients with COPD, admitted in ICU for bronchial infection with few organ failures; Cluster 4 = elderly patients, with several comorbidities and organ failures; Cluster 5 = patients admitted after surgery with a nosocomial infection; Cluster 6 = Young patients, with immunosuppressive disease or therapy, such as AIDS, chronic steroid therapy or hematological malignancy. (DOCX) [file pone.0252793.s013.docx]

S3 Table: Accuracy of the binary tree (performed in training set).

|  | **Truly assigned (Se)** | **Falsely assigned (1-Sp)** | **AUC (IC95%)** |
| --- | --- | --- | --- |
| **Cluster 1** | 1231/1,603 (81%) | 372/2,447 (5%) | 0.82 (0.81-0.82) |
| **Cluster 2** | 118/149 (79%) | 0/3,901 (0%) | 0.87 (0.86-0.87) |
| **Cluster 3** | 231/243 (95%) | 2/3,807 (1%) | 0.97 (0.97-0.98) |
| **Cluster 4** | 746/1,094 (68%) | 431/2,956 (5%) | 0.76 (0.75-0.76) |
| **Cluster 5** | 497/623 (80%) | 143/3,427 (4%) | 0.88 (0.87-0.88) |
| **Cluster 6** | 286/338 (84%) | 70/3,712 (2%) | 0.91 (0.91-0.92) |

*Definition of abbreviations:* Truly assigned = Number of patients correctly assigned as belonging to the cluster according to the total number of patients in the cluster; Falsely assigned = Number of patients incorrectly labeled as belonging to the cluster according to the total number of patients out of the cluster; Se = Sensitivity; Sp = Specificity; AUC = Area under the Receiver Operating Characteristic curve; IC95% = 95% confidence intervals; **Cluster 1** = young patients, without any comorbidities, admitted in ICU for community-acquired pneumonia; **Cluster 2** = young patients, without any comorbidities, admitted in ICU for meningitis or encephalitis; **Cluster 3** = elderly patients with COPD, admitted in ICU for bronchial infection with few organ failures; **Cluster 4** = elderly patients, with several comorbidities and organ failures; **Cluster 5** = patients admitted after surgery with a nosocomial infection; **Cluster 6** = Young patients, with immunosuppressive disease or therapy, such as AIDS, chronic steroid therapy or hematological malignancy.
