# Supplementary material for: Clinical and biological clusters of sepsis patients using hierarchical clustering
Source: PLoS One. 2021 Aug 4;16(8):e0252793. doi: 10.1371/journal.pone.0252793 (PMC8336799; doi:10.1371/journal.pone.0252793)
Supplement: S4 Table — Definition of abbreviations: COPD = chronic obstructive pulmonary disease; HIV = human immunodeficiency virus; AIDS = acquired immune deficiency syndrome; NF GNB: Non-fermentative Gram negative bacilli; MDRO: Multi-drug resistance organisms (refer to vancomycin-resistant enterococci, methicillin-resistant Staphylococcus aureus, extended-spectrum β-lactamase-producing Enterobacteriaceae, AmpC-producing Enterobacteriaceae, Pseudomonas aeruginosa resistant to more than two antimicrobial families, Stenotrophomonas maltophilia); Values in Numbers (%) or median [IQR]. (DOCX) [file pone.0252793.s014.docx]

S4 Table: Characteristics of patients set after assignment in cluster by the cluster analysis (performed in validation set).

| **Variable** | **Cluster 1**  **n=863** | **Cluster 2**  **n=81** | **Cluster 3**  **n=123** | **Cluster 4**  **n=229** | **Cluster 5**  **n=359** | **Cluster 6**  **n=341** |
| --- | --- | --- | --- | --- | --- | --- |
| ***Host characteristics*** |  |  |  |  |  |  |
| Age (years) | 64 [51-76] | 51 [38-62] | 70 [59-77] | 74 [62-81] | 69.5 [59-78] | 58 [47-68] |
| Sex (Male) | 591 (68%) | 49 (60%) | 80 (65%) | 97 (42%) | 225 (63%) | 230 (67%) |
| Weight (kg) | 70 [60-82] | 70.2 [58-76] | 73 [58-87] | 68 [55-80] | 73 [63-85] | 69.5 [57-79.8] |
| Malnutrition | 36 (4%) | 2 (2%) | 5 (4%) | 6 (3%) | 25 (7%) | 24 (7%) |
| Alcohol abuse | 138 (16%) | 14 (17%) | 21 (17%) | 16 (7%) | 44 (12%) | 73 (21%) |
| Not complicated diabetes | 90 (10%) | 6 (7%) | 16 (13%) | 29 (13%) | 37 (10%) | 30 (9%) |
| Complicated diabetes | 25 (3%) | 2 (2%) | 5 (4%) | 20 (9%) | 20 (6%) | 16 (5%) |
| Chronic heart failure | 162 (19%) | 4 (5%) | 20 (16%) | 44 (19%) | 80 (22%) | 46 (13%) |
| Chronic kidney disease | 45 (5%) | 1 (1%) | 4 (3%) | 32 (14%) | 33 (9%) | 47 (14%) |
| Liver cirrhosis | 24 (3%) | 4 (5%) | 3 (2%) | 15 (7%) | 32 (9%) | 80 (23%) |
| COPD | 253 (29%) | 4 (5%) | 100 (81%) | 28 (12%) | 47 (13%) | 30 (9%) |
| Hematological malignancy | 17 (2%) | 5 (6%) | 3 (2%) | 4 (2%) | 5 (1%) | 150 (44%) |
| HIV/AIDS or Transplant | 55 (6%) | 7 (9%) | 1 (1%) | 8 (3%) | 3 (1%) | 39 (11%) |
| Solid tumor | 86 (10%) | 4 (5%) | 5 (4%) | 32 (14%) | 69 (19%) | 57 (17%) |
| Chronic steroid therapy | 38 (4%) | 4 (5%) | 6 (5%) | 18 (8%) | 11 (3%) | 36 (11%) |
| Charlson score | 3 [1-4] | 1 [1-3] | 3 [2-4] | 3 [2-4] | 3 [2-5] | 4 [3-5] |
| ***ICU Admission*** |  |  |  |  |  |  |
| Medical admission | 821 (95%) | 77 (95%) | 115 (93%) | 188 (82%) | 77 (21%) | 331 (97%) |
| Unscheduled surgery | 16 (2%) | 3 (4%) | 4 (3%) | 38 (17%) | 239 (67%) | 7 (2%) |
| Scheduled surgery | 26 (3%) | 1 (1%) | 4 (3%) | 3 (1%) | 43 (12%) | 3 (1%) |
| ***Source of infection*** |  |  |  |  |  |  |
| Pulmonary | 806 (93%) | 11 (14%) | 0 (0%) | 31 (14%) | 43 (12%) | 154 (45%) |
| Bronchial | 0 (0%) | 0 (0%) | 122 (99%) | 0 (0%) | 0 (0%) | 0 (0%) |
| Urinary tract | 3 (0%) | 0 (0%) | 1 (1%) | 201 (88%) | 4 (1%) | 6 (2%) |
| Surgical abdomen | 2 (0%) | 0 (0%) | 0 (0%) | 5 (2%) | 191 (53%) | 5 (1%) |
| Medical abdomen | 3 (0%) | 0 (0%) | 0 (0%) | 6 (3%) | 8 (2%) | 73 (21%) |
| Soft tissues | 11 (1%) | 0 (0%) | 1 (1%) | 4 (2%) | 46 (13%) | 13 (4%) |
| Meningeal encephalitis | 0 (0%) | 81 (100%) | 0 (0%) | 1 (0%) | 0 (0%) | 0 (0%) |
| Miscellaneous sites | 14 (2%) | 5 (6%) | 0 (0%) | 6 (3%) | 56 (16%) | 27 (8%) |
| Unknown site | 24 (3%) | 0 (0%) | 0 (0%) | 7 (3%) | 26 (7%) | 54 (16%) |
| ***Infection micro-organisms*** |  |  |  |  |  |  |
| *Escherichia coli* | 26 (3%) | 1 (1%) | 2 (2%) | 146 (64%) | 76 (21%) | 46 (13%) |
| Other *Enterobacteriaceae* | 47 (5%) | 0 (0%) | 2 (2%) | 67 (29%) | 41 (11%) | 50 (15%) |
| *Pseudomonas* spp. and other NF GNB | 40 (5%) | 0 (0%) | 3 (2%) | 22 (10%) | 24 (7%) | 30 (9%) |
| *Streptococcus pneumoniae* | 111 (13%) | 19 (23%) | 3 (2%) | 4 (2%) | 2 (1%) | 20 (6%) |
| *Enterococcus* and *Streptococcus* | 39 (5%) | 3 (4%) | 2 (2%) | 32 (14%) | 92 (26%) | 31 (9%) |
| *Staphylococcus aureus* | 65 (8%) | 3 (4%) | 1 (1%) | 12 (5%) | 48 (13%) | 21 (6%) |
| Fungus | 7 (1%) | 0 (0%) | 1 (1%) | 4 (2%) | 19 (5%) | 21 (6%) |
| Virus | 24 (3%) | 9 (11%) | 3 (2%) | 1 (0%) | 1 (0%) | 8 (2%) |
| Other pathogens | 165 (19%) | 23 (28%) | 12 (10%) | 14 (6%) | 53 (15%) | 87 (26%) |
| Unknown pathogen | 423 (49%) | 34 (42%) | 99 (80%) | 14 (6%) | 135 (38%) | 111 (33%) |
| Bacteriemia | 51 (6%) | 10 (12%) | 2 (2%) | 94 (41%) | 60 (17%) | 91 (27%) |
| Nosocomial | 220 (25%) | 20 (25%) | 24 (20%) | 146 (64%) | 195 (54%) | 116 (34%) |
| MDRO | 40 (5%) | 2 (2%) | 6 (5%) | 67 (29%) | 56 (16%) | 29 (9%) |
| ***Host response*** |  |  |  |  |  |  |
| Myocardial dysfunction | 158 (18%) | 12 (15%) | 17 (14%) | 58 (25%) | 77 (21%) | 83 (24%) |
| Cardiac arrest before admission | 51 (6%) | 2 (2%) | 0 (0%) | 9 (4%) | 7 (2%) | 24 (7%) |
| Hyperglycemia (>11 mmol/l) | 119 (14%) | 12 (15%) | 15 (12%) | 37 (16%) | 52 (14%) | 72 (21%) |
| Hypoglycemia (<3 mmol/l) | 12 (1%) | 2 (2%) | 0 (0%) | 6 (3%) | 7 (2%) | 37 (11%) |
| Body temperature (°C) | 38.3 [37.6-39] | 38.5 [37.6-39.2] | 38 [37.4-38.5] | 38.2 [37.4-38.8] | 38 [37.5-38.6] | 38.5 [37.6-39.3] |
| New atrial fibrillation | 104 (12%) | 9 (11%) | 22 (18%) | 25 (11%) | 44 (12%) | 87 (26%) |
| Recurrent atrial fibrillation | 55 (6%) | 0 (0%) | 7 (6%) | 22 (10%) | 27 (8%) | 21 (6%) |
| Heart rate (beats/min) | 115 [100-132] | 120 [100-136] | 118 [100-136] | 115 [100-131] | 116 [100-131] | 124 [110-143] |
| Respiratory rate (breaths/min) | 26 [20-33] | 22 [19-30] | 28 [20-35] | 24 [20-30] | 20 [16-25] | 27 [22-33] |
| Sodium blood level (mmol/l) | 138 [134-142] | 136 [133-141] | 138 [134-140] | 137 [132-142] | 137 [133-141] | 136 [132-140] |
| Potassium blood level (mmol/l) | 4 [3.6-4.4] | 3.6 [3.1-4] | 4.2 [3.8-4.7] | 3.9 [3.3-4.6] | 4.2 [3.7-4.8] | 3.9 [3.3-4.7] |
| Bicarbonate blood level (mmol/l) | 23 [19-26] | 21 [17-24] | 26 [22-31] | 18 [14-22] | 19 [15-23] | 18 [14-22] |
| Hematocrit (%) | 36 [31-41] | 35 [32-41] | 39 [34-44] | 32 [27-36] | 31 [27-36] | 27 [23-32] |
| Prothrombin time (%) | 73 [61-86] | 71 [58-87] | 79 [62.5-91] | 60 [44-73] | 61 [48-71] | 53 [39-64] |
| Leukocytes (x10^3^/mm3), | 12,8 [8,5-18,2] | 12,4 [7,7-19,2] | 12,3 [9,4-16,9] | 16,0 [10,7-23,9] | 14,0 [8,2-20,1] | 5,4 [0.9-13,3] |
| Fluid replacement >50 ml/kg | 172 (20%) | 20 (25%) | 8 (7%) | 52 (23%) | 80 (22%) | 92 (27%) |
| ***Organ failure*** |  |  |  |  |  |  |
| Vasopressor at admission | 436 (51%) | 40 (49%) | 34 (28%) | 150 (66%) | 259 (72%) | 247 (72%) |
| Glasgow Coma Score | 14 [8-15] | 8 [6-13] | 15 [13-15] | 15 [10-15] | 15 [13-15] | 15 [7-15] |
| Creatinine level (µmol/l) | 90 [68-131] | 89.5 [64.5-131] | 85 [65-113] | 158 [105-260] | 120.5 [79-203.5] | 129.5 [85-223] |
| Platelets count (x10^3^/mm^3^) | 217 [158-293] | 177 [115-227] | 234 [169-299] | 168 [110-243] | 220 [144-334] | 67 [31-128] |
| PaO2/FiO2 ratio (mmHg) | 209 [130-323] | 341 [209-460] | 215 [139-327] | 260 [175-438] | 255 [166-378] | 238 [122-438] |
| Bilirubin level (mmol/l) | 11 [7-17] | 14 [8-24] | 10 [6-15] | 13 [8-24] | 16 [10.4-28] | 23 [11-43.9] |
| Blood lactate level (mmol/l) | 1.7 [1.2-2.8] | 1.8 [1.3-2.9] | 1.4 [1-1.9] | 2.3 [1.5-4.2] | 2.1 [1.2-3.5] | 2.9 [1.5-6.5] |

*Definition of abbreviations:* COPD = chronic obstructive pulmonary disease; HIV = human immunodeficiency virus; AIDS = acquired immune deficiency syndrome; NF GNB: non-fermentative Gram negative bacilli; MDRO: multi-drug resistance organisms (refer to vancomycin-resistant enterococci, methicillin-resistant *Staphylococcus aureus*, extended-spectrum β-lactamase-producing *Enterobacteriaceae*, AmpC-producing *Enterobacteriaceae*, *Pseudomonas aeruginosa* resistant to more than two antimicrobial families, *Stenotrophomonas maltophilia*); Values in Numbers (%) or median [IQR].
