# Supplementary material for: Clinical and biological clusters of sepsis patients using hierarchical clustering
Source: PLoS One. 2021 Aug 4;16(8):e0252793. doi: 10.1371/journal.pone.0252793 (PMC8336799; doi:10.1371/journal.pone.0252793)
Supplement: S5 Table — Definition of abbreviations: Truly assigned = Number of patients correctly assigned as belonging to the cluster according to the total number of patients in the cluster; Falsely assigned = Number of patients incorrectly labeled as belonging to the cluster according to the total number of patients out of the cluster; Se = Sensitivity; Sp = Specificity; AUC = Area under the Receiver Operating Characteristic curve; IC95% = 95% confidence intervals; Cluster 1 = young patients, without any comorbidities, admitted in ICU for community-acquired pneumonia; Cluster 2 = young patients, without any comorbidities, admitted in ICU for meningitis or encephalitis; Cluster 3 = elderly patients with COPD, admitted in ICU for bronchial infection with few organ failures; Cluster 4 = elderly patients, with several comorbidities and organ failures; Cluster 5 = patients admitted after surgery with a nosocomial infection; Cluster 6 = Young patients, with immunosuppressive disease or therapy, such as AIDS, chronic steroid therapy or hematological malignancy. (DOCX) [file pone.0252793.s015.docx]

S5 Table: Accuracy of the binary tree (performed in validation set).

|  | **Truly assigned (Se)** | **Falsely assigned (1-Sp)** | **AUC (IC95%)** |
| --- | --- | --- | --- |
| **Cluster 1** | 639/863 (74%) | 136/1,133 (12%) | 0.81 (0.80-0.81) |
| **Cluster 2** | 66/81 (81%) | 1/1,915 (1%) | 0.90 (0.90-0.91) |
| **Cluster 3** | 114/123 (92%) | 0/1,873 (0%) | 0.96 (0.96-0.97) |
| **Cluster 4** | 166/229 (72%) | 400/1,767 (23%) | 0.75 (0.74-0.75) |
| **Cluster 5** | 246/359 (69%) | 59/1,637 (3%) | 0.82 (0.82-0.83) |
| **Cluster 6** | 146/341 (43%) | 23/1,665 (1%) | 0.70 (0.70-0.71) |

*Definition of abbreviations:* Truly assigned = Number of patients correctly assigned as belonging to the cluster according to the total number of patients in the cluster; Falsely assigned = Number of patients incorrectly labeled as belonging to the cluster according to the total number of patients out of the cluster; Se = Sensitivity; Sp = Specificity; AUC = Area under the Receiver Operating Characteristic curve; IC95% = 95% confidence intervals; **Cluster 1** = young patients, without any comorbidities, admitted in ICU for community-acquired pneumonia; **Cluster 2** = young patients, without any comorbidities, admitted in ICU for meningitis or encephalitis; **Cluster 3** = elderly patients with COPD, admitted in ICU for bronchial infection with few organ failures; **Cluster 4** = elderly patients, with several comorbidities and organ failures; **Cluster 5** = patients admitted after surgery with a nosocomial infection; **Cluster 6** = Young patients, with immunosuppressive disease or therapy, such as AIDS, chronic steroid therapy or hematological malignancy.
