# Supplementary material for: Clinical and biological clusters of sepsis patients using hierarchical clustering
Source: PLoS One. 2021 Aug 4;16(8):e0252793. doi: 10.1371/journal.pone.0252793 (PMC8336799; doi:10.1371/journal.pone.0252793)
Supplement: S6 Table — Definition of abbreviations: COPD = chronic obstructive pulmonary disease; HIV = human immunodeficiency virus; AIDS = acquired immune deficiency syndrome; NF GNB: Non-fermentative Gram negative bacilli; MDRO: Multi-drug resistance organisms (refer to vancomycin-resistant enterococci, methicillin-resistant Staphylococcus aureus, extended-spectrum β-lactamase-producing Enterobacteriaceae, AmpC-producing Enterobacteriaceae, Pseudomonas aeruginosa resistant to more than two antimicrobial families, Stenotrophomonas maltophilia); Values in Numbers (%) or median [IQR]. (DOCX) [file pone.0252793.s016.docx]

S6 Table : Characteristics of patients after excluding the oldest data (i.e., admission before 2008) (performed in training set).

| **Variable** | **Cluster 1**  **n=847** | **Cluster 2**  **n=70** | **Cluster 3**  **n=107** | **Cluster 4**  **n=519** | **Cluster 5**  **n=283** | **Cluster 6**  **n=216** |
| --- | --- | --- | --- | --- | --- | --- |
| ***Host characteristics*** |  |  |  |  |  |  |
| Age (years) | 63 [49-75] | 59 [36-68] | 71 [59-77] | 72 [62-79] | 66 [57-77] | 58 [44-65] |
| Sex (Male) | 555 (66%) | 34 (49%) | 63 (59%) | 277 (53%) | 187 (66%) | 135 (63%) |
| Weight (kg) | 70 [58-83] | 65 [57-80] | 72 [60-90] | 72 [62-83] | 74 [64-88] | 72 [62-82] |
| Malnutrition | 79 (9%) | 4 (6%) | 7 (7%) | 35 (7%) | 25 (9%) | 15 (7%) |
| Alcohol abuse | 162 (19%) | 5 (7%) | 18 (17%) | 78 (15%) | 43 (15%) | 17 (8%) |
| Not complicated diabetes | 95 (11%) | 10 (14%) | 18 (17%) | 111 (21%) | 42 (15%) | 21 (10%) |
| Complicated diabetes | 22 (3%) | 4 (6%) | 8 (7%) | 76 (15%) | 24 (8%) | 3 (1%) |
| Chronic heart failure | 109 (13%) | 7 (10%) | 20 (19%) | 187 (36%) | 87 (31%) | 17 (8%) |
| Chronic kidney disease | 36 (4%) | 5 (7%) | 5 (5%) | 98 (19%) | 42 (15%) | 22 (10%) |
| Liver cirrhosis | 48 (6%) | 3 (4%) | 3 (3%) | 85 (16%) | 40 (14%) | 13 (6%) |
| COPD | 215 (25%) | 5 (7%) | 88 (82%) | 95 (18%) | 33 (12%) | 19 (9%) |
| Hematological malignancy | 29 (3%) | 5 (7%) | 2 (2%) | 9 (2%) | 10 (4%) | 179 (83%) |
| HIV/AIDS or Transplant | 39 (5%) | 8 (11%) | 0 (0%) | 29 (6%) | 12 (4%) | 36 (17%) |
| Solid tumor | 102 (12%) | 4 (6%) | 8 (7%) | 101 (19%) | 68 (24%) | 34 (16%) |
| Chronic steroid therapy | 38 (4%) | 7 (10%) | 6 (6%) | 32 (6%) | 18 (6%) | 20 (9%) |
| Charlson score | 3 [1-4] | 2 [1-3] | 3 [2-4] | 4 [3-6] | 3 [2-5] | 3 [3-5] |
| ***ICU Admission*** |  |  |  |  |  |  |
| Medical admission | 805 (95%) | 68 (97%) | 104 (97%) | 462 (89%) | 53 (19%) | 211 (98%) |
| Unscheduled surgery | 18 (2%) | 1 (1%) | 2 (2%) | 43 (8%) | 206 (73%) | 5 (2%) |
| Scheduled surgery | 24 (3%) | 1 (1%) | 1 (1%) | 14 (3%) | 24 (8%) | 0 (0%) |
| ***Source of infection*** |  |  |  |  |  |  |
| Pulmonary | 737 (87%) | 13 (19%) | 0 (0%) | 172 (33%) | 22 (8%) | 22 (8%) |
| Bronchial | 0 (0%) | 0 (0%) | 107 (100%) | 0 (0%) | 0 (0%) | 0 (0%) |
| Urinary tract | 15 (2%) | 0 (0%) | 0 (0%) | 198 (38%) | 3 (1%) | 3 (1%) |
| Surgical abdomen | 1 (0%) | 0 (0%) | 0 (0%) | 2 (0%) | 185 (65%) | 185 (65%) |
| Medical abdomen | 21 (2%) | 0 (0%) | 0 (0%) | 37 (7%) | 6 (2%) | 6 (2%) |
| Soft tissues | 11 (1%) | 1 (1%) | 0 (0%) | 31 (6%) | 50 (18%) | 50 (18%) |
| Meningeal encephalitis | 0 (0%) | 70 (100%) | 0 (0%) | 0 (0%) | 0 (0%) | 0 (0%) |
| Miscellaneous sites | 27 (3%) | 4 (6%) | 3 (3%) | 46 (9%) | 12 (4%) | 12 (4%) |
| Unknown site | 26 (3%) | 0 (0%) | 0 (0%) | 64 (12%) | 9 (3%) | 9 (3%) |
| ***Infection micro-organisms*** |  |  |  |  |  |  |
| *Escherichia coli* | 22 (3%) | 1 (1%) | 2 (2%) | 214 (41%) | 49 (17%) | 49 (17%) |
| Other *Enterobacteriaceae* | 55 (6%) | 0 (0%) | 0 (0%) | 113 (22%) | 35 (12%) | 35 (12%) |
| *Pseudomonas* spp. and other NF GNB | 51 (6%) | 0 (0%) | 7 (7%) | 39 (8%) | 19 (7%) | 19 (7%) |
| *Streptococcus pneumoniae* | 126 (15%) | 20 (29%) | 1 (1%) | 12 (2%) | 1 (0%) | 1 (0%) |
| *Enterococcus* and *Streptococcus* | 32 (4%) | 4 (6%) | 4 (4%) | 70 (13%) | 81 (29%) | 81 (29%) |
| *Staphylococcus aureus* | 80 (9%) | 2 (3%) | 1 (1%) | 62 (12%) | 16 (6%) | 16 (6%) |
| Fungus | 8 (1%) | 1 (1%) | 0 (0%) | 15 (3%) | 13 (5%) | 13 (5%) |
| Virus | 45 (5%) | 9 (13%) | 1 (1%) | 12 (2%) | 0 (0%) | 0 (0%) |
| Other pathogens | 201 (24%) | 25 (36%) | 12 (11%) | 57 (11%) | 52 (18%) | 52 (18%) |
| Unknown pathogen | 374 (44%) | 19 (27%) | 83 (78%) | 89 (17%) | 121 (43%) | 121 (43%) |
| Bacteriemia | 70 (8%) | 15 (21%) | 2 (2%) | 174 (34%) | 41 (14%) | 41 (14%) |
| Nosocomial | 207 (24%) | 13 (19%) | 19 (18%) | 155 (30%) | 112 (40%) | 112 (40%) |
| MDRO | 50 (6%) | 2 (3%) | 4 (4%) | 64 (12%) | 23 (8%) | 23 (8%) |
| ***Host response*** |  |  |  |  |  |  |
| Myocardial dysfunction | 113 (13%) | 9 (13%) | 14 (13%) | 197 (38%) | 70 (25%) | 26 (12%) |
| Cardiac arrest before admission | 41 (5%) | 3 (4%) | 4 (4%) | 66 (13%) | 11 (4%) | 8 (4%) |
| Hyperglycemia (>11 mmol/l) | 175 (21%) | 17 (24%) | 32 (30%) | 192 (37%) | 68 (24%) | 64 (30%) |
| Hypoglycemia (<3 mmol/l) | 16 (2%) | 5 (7%) | 5 (5%) | 54 (10%) | 5 (2%) | 7 (3%) |
| Body temperature (°C) | 38.2 [37.6-39] | 38.4 [37.4-39.4] | 37.6 [37.3-38.1] | 37.9 [37.4-38.5] | 37.9 [37.3-38.6] | 38.8 [38-39.5] |
| New atrial fibrillation | 114 (13%) | 9 (13%) | 9 (8%) | 82 (16%) | 47 (17%) | 52 (24%) |
| Recurrent atrial fibrillation | 54 (6%) | 3 (4%) | 12 (11%) | 81 (16%) | 28 (10%) | 8 (4%) |
| Heart rate (beats/min) | 114 [101-130] | 109 [95-130] | 111 [99-125] | 115 [99-136] | 118 [100-135] | 126 [110-143] |
| Respiratory rate (breaths/min) | 27 [22-33] | 24 [20-32] | 25 [20-31] | 25 [20-32] | 22 [17-27] | 30 [25-36] |
| Sodium blood level (mmol/l) | 138 [135-142] | 137 [134-142] | 140 [137-142] | 138 [133-142] | 137 [134-142] | 136 [133-140] |
| Potassium blood level (mmol/l) | 3.9 [3.5-4.4] | 3.8 [3.3-4.3] | 4.3 [3.8-4.9] | 4.1 [3.5-5] | 4.2 [3.7-4.9] | 3.7 [3.3-4.2] |
| Bicarbonate blood level (mmol/l) | 22 [19-26] | 21 [16-23] | 27 [22-34] | 18 [14-21.3] | 18.8 [15-22] | 19 [15.3-23] |
| Hematocrit (%) | 35 [30-40] | 35 [30-40] | 38 [33-44] | 32 [27-36] | 32 [28-37] | 25 [22-28] |
| Prothrombin time (%) | 75 [61-85.4] | 74 [62-85.7] | 81 [59-92] | 57 [40-70] | 60 [48-70] | 59 [48.5-71.5] |
| Leukocytes (x10^3^/mm3), | 12.3 [8.2-1.7] | 12.3 [7.6-2.1] | 12.5 [8.9-1.6] | 15.1 [9.4-2.1] | 13.8 [8.2-2.0] | 12.0 [0.3-4.8] |
| Fluid replacement >50 ml/kg | 156 (18%) | 16 (23%) | 7 (7%) | 99 (19%) | 79 (28%) | 40 (19%) |
| ***Organ failure*** |  |  |  |  |  |  |
| Vasopressor at admission | 439 (52%) | 34 (49%) | 36 (34%) | 393 (76%) | 210 (74%) | 125 (58%) |
| Glasgow Coma Score | 14 [7-15] | 9 [6-13] | 15 [10-15] | 14 [6-15] | 15 [10-15] | 15 [15-15] |
| Creatinine level (µmol/l) | 86 [63-123] | 85 [69-121] | 79 [56-117] | 165 [102-270] | 125 [80-221] | 115 [74-188] |
| Platelets count (x10^3^/mm^3^) | 221 [153-291] | 212 [116-287] | 230 [182-295] | 179 [112-264] | 205 [131-289] | 45 [27-80] |
| PaO2/FiO2 ratio (mmHg) | 193 [120-309] | 322 [224-495] | 223 [149-296] | 217 [133-341] | 264 [164-379] | 326 [187-500] |
| Bilirubin level (mmol/l) | 11 [7-17] | 13 [8-22] | 9 [5-15] | 16 [9-30] | 17 [10-29] | 18 [10-36] |
| Blood lactate level (mmol/l) | 1.8 [1.2-2.8] | 2.2 [1.3-3.6] | 1.5 [1.2-2.7] | 2.7 [1.6-5] | 2.2 [1.5-3.7] | 2.3 [1.5-4.4] |

*Definition of abbreviations:* COPD = chronic obstructive pulmonary disease; HIV = human immunodeficiency virus; AIDS = acquired immune deficiency syndrome; NF GNB: non-fermentative Gram negative bacilli; MDRO: multi-drug resistance organisms (refer to vancomycin-resistant enterococci, methicillin-resistant Staphylococcus aureus, extended-spectrum β-lactamase-producing Enterobacteriaceae, AmpC-producing Enterobacteriaceae, Pseudomonas aeruginosa resistant to more than two antimicrobial families, Stenotrophomonas maltophilia); Values in Numbers (%) or median [IQR].
