# Supplementary material for: Clinical and biological clusters of sepsis patients using hierarchical clustering
Source: PLoS One. 2021 Aug 4;16(8):e0252793. doi: 10.1371/journal.pone.0252793 (PMC8336799; doi:10.1371/journal.pone.0252793)
Supplement: S7 Table — (DOCX) [file pone.0252793.s017.docx]

S7 Table : Number of patients included and median of admission in each center.

| **ICU number** | **Effectif** | **Percent(%)** | **Median of admission [IQR]** |
| --- | --- | --- | --- |
| **1** | 1514 | 25.04 | 2006 [2001-2009] |
| **2** | 1479 | 24.46 | 2009 [2008-2010] |
| **3** | 221 | 3.66 | 2007 [2003-2008] |
| **4** | 300 | 4.96 | 2008 [2006-2010] |
| **5** | 252 | 4.17 | 2003 [1998-2008] |
| **6** | 23 | 0.38 | 2010 [2009-2010] |
| **7** | 187 | 3.09 | 2012 [2010-2014] |
| **8** | 103 | 1.70 | 2006 [2004-2008] |
| **9** | 2 | 0.03 | 2006 [2006-2006] |
| **10** | 118 | 1.95 | 2013 [2012-2013] |
| **11** | 2 | 0.03 | 2009 [2009-2009] |
| **12** | 65 | 1.08 | 2007 [2001-2007] |
| **13** | 839 | 13.88 | 2008 [2006-2013] |
| **14** | 300 | 4.96 | 2007 [2004-2007] |
| **15** | 59 | 0.98 | 2012 [2011-2012] |
| **16** | 52 | 0.86 | 2011 [2011-2013] |
| **17** | 129 | 2.13 | 2002 [2001-2003] |
| **18** | 27 | 0.45 | 2004 [2003-2005] |
| **19** | 362 | 5.99 | 1999 [1998-2001] |
| **20** | 12 | 0.20 | 2012 [2011-2012] |
