# Supplementary material for: Clinical and biological clusters of sepsis patients using hierarchical clustering
Source: PLoS One. 2021 Aug 4;16(8):e0252793. doi: 10.1371/journal.pone.0252793 (PMC8336799; doi:10.1371/journal.pone.0252793)
Supplement: Attachment — Submitted filename: Reviewers.docx [file pone.0252793.s019.docx]

**Reviewer #1:** **The heterogeneity of sepsis patients and their potential grouping into clusters are among the hot topics in epidemiological sepsis research and the design of better clinical sepsis trials. Therefore it is very laudable to apply this approach to the OUTCOMEREA database, especially as it is representative of all, unselected sepsis patients admitted to the ICU in an European setting and therefore nicely adds to previous studies with datasets from the US or from clinical trials with patient selection. Even though I lack the expertise to judge the details of the methodology the chosen approach is discussed in detail and the results are presented in great detail and precise language. The resulting clusters correspond well to my clinical experience and corresponding patients are probably recognized by all intensivists.**

**My only concern, apart from my inability to judge the methodological details, is the availability of data.**

We thank the reviewer for their thoughtful review and comments. Please see our point-by-point responses below.

**#R1.1: The authors claim that “All relevant data are within the manuscript and its Supporting Information files”, but this is not correct. According to the PLOS data policy the individual patient data used in the analysis should be available. If this is impossible due to legal restrictions the unavailability of the data needs to be stated and explained. Aside from this I see no need for further revisions.**

**R1.1:** In the first submission, the data were provided in two files, after random separation into "Test set" and "Training set". We have corrected this. One raw data file including all patients before assignment and before imputation is now available as an additional file.

**Reviewer #2: The authors present a secondary analysis of data obtained with the aim to identify phenotypes among patients with sepsis. To accomplish this, they used an existing multi-center French ICU database including patients treated between 1997 and 2015 (or 2005?). Patients with sepsis according to sepsis-3 were included in the analysis. They conducted a cluster-analysis in a randomly selected training sample involving 63 clinical and biological variables available at ICU admission and thereby identified 6 phenotypes of patients with sepsis. These phenotypes showed differences in patient outcomes (mainly short- and long-term mortality). By using a binary tree analysis, they developed a simple set of rules to identify patients belonging to one of the clusters. Applying these rules in a randomly chosen training data set (taken from the same database) they tested if the pattern of distributions of variables between clusters and the relations with patient’s outcomes were replicable, which was mostly the case.**

**A better understanding of distinct subgroups of patients with sepsis is of high importance for developing new and improved diagnostics and treatments. The reported analyses seem professionally conducted and were applied to a large data set. My major concern is, that the authors “started from scratch” in a fully exploratory approach without trying to replicate phenotypes that were reported previously (e.g. Seymour et al, Knox et al, Garlund et al). Thereby, it remains unclear, if they find different phenotypes because of a different sample, different variables used for clustering, or different clustering methods. In general, the authors should strive to conduct empirical comparisons between existing phenotypes and the clusters that they found within their data. If this is done, the paper could add a lot more knowledge. If we consider this paper as a “role model” for future research regarding phenotypes in sepsis, then we should prevent a “fully exploratory” approach of every new paper, since this would result in large number of sets of different phenotypes only loosely connectible across studies.**

We agree. The multiplication of clustering models in different cohorts increase the risk of identifying clusters that are not comparable. An external comparison and validation of clusters in other external cohort will be of key interest.

In their editorial (PMID: 31062051), J.C. Forte *et al*. proposed two steps to compare and validate a cluster analysis: 1) train a supervised classifier on sub-sample of the dataset to assess its accuracy and 2) train the supervised classifier on an external dataset with the same variables seems like a better approach.

As we develop it in the answer R2.9.R, the variables are different in the published clustering studies. So clusters which are currently published are complementary and cannot be compared. In future, researches should be converge to a database including all these variables.

We add in the discussion section: “future studies are needed to aggregate different data in order to combine clinical data, host genomics, transcriptomic responses and cytokines, and using data science approaches accounting for longitudinal data”.

**Some more detailed recommendations for improving the paper:**

We thank the reviewer for their thoughtful review and comments. Please see our point-by-point responses below.

**#R2.1: Classify this study as a secondary data analysis early in the text (in the abstract and early in the methods), discuss possible shortcomings of using existing data, that were not gathered for the purpose of the study.**

**R.2.1.R:** We thank the reviewer for his/her comment.

**What we changed:**

1) We add the sentence “retrospective analysis of a prospective database” to the abstract and to the method section.

2) In Discussion, we modified the Limitation part by adding: “Due to the retrospective analysis of a prospective database some limitations must be acknowledged for this study. First, unsupervised analysis is not unbiased because only the data available within the database can be explored, and only the data that are known or thought to be important are entered (PMID: 31593265). Some data would have been necessary, in particular data about clinical management or specific therapy (PMID: 30563548)”.

**#R2.2: Refer to an appropriate guideline for your study and structure the paper accordingly (e.g. STROBE)**

**R.2.2.R:** In line with the STROBE Statement, several changes in the structure of the article have been made in particular in the methods and discussion sections.

**#R2.3: The data have been obtained across a long time period (1997 to 2015- one time it is stated 2005), describe and discuss possible changes in patient population and sepsis management that might have influence on results.**

**R.2.3.R:** We agree with the reviewer comment. To account for this possible bias in results, a new analysis was performed by excluding patients for whom the data were collected before 2008 median year of admission in our dataset). The new analysis was performed with n=2,042 patients and conducted to the identification of six clusters with similar features.

**What we changed:** The results of main feature associations and mortality of the clusters identified in this new analysis, without the oldest data, were added into the Supplemental Digital Content as a sensitivity analysis (S8 Table).

**#R2.4: It is unclear how patients with sepsis where identified: by clinical review of case-data? By an automatic database query? How often was SOFA Score measured? Daily? More than once a day? Were patients included having sepsis at admission or getting sepsis during their stay?**

**R.2.4.R**: We thank the reviewer for his/her comment; indeed, the study population, definition of infection and definition of sepsis requires further clarification.

**Study population:** All patients included were those admitted in ICU for sepsis and septic shock. Patients with ICU-acquired infections were excluded.

**Definition of infection:** The presence or absence of infection was recorded prospectively by clinical physicians according to the standard definitions developed by the Centers for Disease Control and Prevention and recently updated (PMID: 16003060).

**Definition of sepsis:** According to the new Sepsis 3.0 definition (PMID 26903338), sepsis was defined as a life-threatening organ dysfunction, identified by an increase by two points or more of the SOFA score, associated with an infection. Patients were included in the OUTCOMEREA database prior to this new definition. Accordingly, increase in the SOFA score was calculated retrospectively. The maximum SOFA score measured on the first day of ICU stay was used. Prior to admission in ICU, the baseline SOFA score can be considered to be at zero. The baseline SOFA score of patients with chronic renal replacement therapy was assumed at 4 points.

**What we changed:** The method section was modified to include these definitions.

**#R2.5:** **How was it validated that the increase in SOFA was due to infection? How was predictive accuracy of identification of patients with sepsis assured (sensitivity, specificity). If the identification of patients with sepsis might be prone to selection bias, this needs to be discussed.**

**R2.5.R:** We thank the reviewer for his/her comment and we agree with the need of a gold standard to have an accurate diagnosis of the sepsis. However, at our knowledge, there is no reference diagnostic method for sepsis. We consider as reference the new definition of sepsis proposed by M. Singer in 2016 (PMID 26903338), including the increase in SOFA score.

**What we changed:** We added the sentence as follow in the discussion section: “Due to the absence of gold-standard method for sepsis diagnosis, we cannot exclude a selection bias for patients with sepsis. However, due to the high quality database, we can ensure that all selected patients have a sepsis diagnosis confirmed by a clinician.”

**#R2.6: Additionally, it is not clear, when the characteristics used for clustering were measured during the ICU stay- at admission? In a defined period of time before or after the onset of sepsis? This is essential for interpretation and comparison of the study results.**

**R2.6.R:** We thank the reviewer for his/her comment and to help us to improve the clarity of our manuscript. All patients included where those admitted in ICU with a diagnosis of sepsis or septic shock. All data included in the clustering were measured at ICU admission or within the first day of ICU stay.

**What we changed:** Details were added in the method section and in title of the Table 1.

**#R2.7:** **Justify the method used for handling missing data, why was multiple imputation not used? Give a reference that proves Markov Chain Monte Carlo a valid method for handling missing data.**

**R2.7.R:** We thank again the reviewer for his/her comment and to help us to improve the clarity of our manuscript. Markov chain Monte Carlo was an algorithm for multiple imputations implemented in SAS 9.4.

**What we changed:** Reference (PMID: 10347857) and the following sentence: “For variables with less than 20% of missing values, we performed a multiple imputation of missing data using Markov Chain Monte Carlo” was added in the methods section.

**#R2.8:** **The results are presented in numerous (supplementary) tables and figures. This makes it really burdensome for the reader to focus on important results. Consider using mostly graphical presentations as has been done in previous publications (e.g. Seymour et al, Knox et al) and bringing small bits of result presentations that are now separate into larger chunks of information that allow an overview (e.g. information of Fig S7-S11 and the corresponding descriptive tables could be shown in a single panel figure comparable to Seymour et al Fig. 2).**

**R2.8.R:** We thank the reviewer for his/her comment.

**What we changed:** As suggested, figures S7 to S11 and the associated tables have been merged in new Figure S7 and S5 Table.

**#R2.9:** **In line 93-96 referring to Seymour et al the authors state that comparison of cluster analysis on different cohorts is essential. This comparison should be done empirically within the paper: Authors should try to replicate existing phenotypes in their own data (especially Seymour et al. phenotypes). If existing phenotypes prove not to be replicable in new data (despite using comparable methods regarding set of variables, inclusion criteria, statistical methods) - this is an important result that needs to be discussed. If “Seymour” clusters are approximately replicable, then authors should do empirical comparisons with their newly identified clusters.**

**R2.9.R:** We agree with the Reviewer’ comment in that empirical comparison could help to interpret differences in clustering and would allow for external validity. However, several variables used in Seymour's clustering are related to inflammation and are not available in the Outcomerea database. Conversely, clinical data related to comorbidities and infections, pathogens, bacterial resistance and infection sites presents in the Outcomerea database were not used in Seymour's study and can be considered as essential for describing sepsis phenotypes. Therefore, it is impossible to perform empirical comparisons between our results and Seymour’s ones.

**What we changed:** As the Reviewer, we think that this point is instrumental for future researches, and we add in the discussion section: “future studies are needed to aggregate different data in order to combine clinical data, host genomics, transcriptomic responses and cytokines, and using data science approaches accounting for longitudinal data”.

**#R2.10: The paper is partly not correctly structured: part of results tables and figures are already referred to in the methods section (and I believe not completely in order), also part of the results is mentioned in the text of the methods section. Methods and results should be clearly separated and no result should be referred to already in the methods section.**

**R2.10.R:** We thank the reviewer for his/her comment which will help us to improve our manuscript.

**What we changed:** According to this comment, references to tables and figures have been corrected and associated with the results section. Only Table 1 describing the variables used in the cluster analysis and S1 Fig. describing the schematic of study has been kept in the method section.

**#R2.11:** **The purpose of the binary tree analysis is not really made clear. Please give better explanation. Wouldn’t a completely new replication of the cluster analysis in a second, independent data set be a better validation?**

**R2.11.R:** Concerning utility of CART method: Although presenting some weaknesses, CART is a simple tool, which allows allocating a new patient to a cluster, which is easily understandable by clinicians, and which could be easily used in future studies.

We agree, reproducing the cluster analysis in the validation set also assesses the reproducibility of our method.

**What we changed:** We performed a new cluster analysis using MCA and HCA in the validation set (S5 Table). Difference in results obtained were assessed using Se and Sp (S7 Table).

**#R2.12: If training and validation set are random subsamples of the same sample, then there is no true validation done. As long as the two samples are large enough (thereby excluding sampling bias) they will mostly show very similar results. Is there a better way to define a training and a validation data set? Could the authors use additional data (e.g. trial data) to validate their phenotypes? If not, this should be discussed as a limitation.**

**R2.12.R:** We agree that external validation cohort is critical; it will be an upcoming study for our team. This information was added to the discussion section as follow: ”Finally, the training set and the validation set come from the same database. A validation with an external dataset such as SENECA, GenIMS, ACCESS, PROWES or ProCESS cohorts used in Seymour study would provide more robust results.”.

**#R2.13:** **To date, several methods have been used to identify phenotypes: hierarchical clustering done by the authors of this study, k-means clustering, latent class analysis (Seymour et al, Garlund et al), latent profile analysis (Zhang et al), self-organizing maps (Zhang et al). Since clustering results are highly dependent on the used variables and methods, sensitivity analyses should involve repeating the analysis with different methods, and with excluding and including different sets of variables.**

**R2.13.R:** We thank the reviewer for his/her comment. Although we agree with the need to compare several cluster analyses, here it was not our objective and beyond the scope of our work. Indeed, we want to assess the interest of identify cluster inside a national cohort by using an unsupervised method, and to verify that 1) these clusters are clinically different and 2) these clusters are associated to potential outcomes. Moreover, there is no common standard for clustering validation (PMID 31062051).

**What we changed:** We have chosen to evaluate the stability of our method after exclusion of the oldest data and to propose an improvement in our results, as suggested in #R2.11 (S8 Table). As specified in R2.11.R, we also provided a cluster analysis in the validation set (S5 Table).

**#R2.14:** **Why was an MCA conducted before the cluster analysis? Especially since the from 63 variables the information was just reduced to 52 dimensions? How could this bias the results? How sensitive are the results to omitting the MCA or specifying it in different ways?**

**R2.14.R:** We thank the reviewer for his/her comment. To be more accurate, MCA reduced information of 137 modalities of 63 variables to 52 dimensions. The reduction of the dimension will not bias the results; due to the fact that coordinates will be obtained for each patient which summarizes the main part of the information. MCA cannot be omitted from this method. The association of these two methods allows also accounting continuous variables in the HC. By avoiding MCA, qualitative variables cannot be considered in the clustering, and another pattern of cluster will be identified due to a loss of information.

**What we changed:** Sentence was modified in the introduction section as follow : “This clustering method has two advantages; it limits the noise in the data set without inducing bias by reducing the variables in coordinates obtained for each patient which summarizes the main part of the information and does not require defining *a priori* the number of clusters.”.

**#R2.15:** **Give an explanation why the specific method for clustering was chosen. Seymour et al chose their method of k-means clustering based on empirical arguments (“OPTICS plot”).**

**R2.15.R:** As specified in the introduction section, there is no common guideline for clustering. In other words, different methods exist, each of them with advantages and pitfalls (PMID: 26389570). For our study, the choice of HC associated with dimension reduction is based on the fact that it has already been applied and acknowledged in different medical disciplines (PMID 27314230, 19892860, 22962314, 23236428, 25898956). Although this method presents some limitations, the advantages are 1) the data reduction of the dataset [patients X variables] in dataset [patients X dimensions], 2) it is an unsupervised classification method and it does not require to define *a priori* the number of clusters, and 3) the dendrogram in output can help to choose the final number of clusters.

We have added in the method section: “Due to the absence of recommendations, we have empirically chosen combination of MCA and HC”

**#R2.16:** **Describe the data base used: what was the original purpose of gathering the data? How could this bias the results of the secondary analysis?**

**R2.16.R:** As suggested and in accordance with the strobe statement this database information has been added to the methods section.

**#R2.17:** **How much of the variability in the data is actually explained by the clusters and how much variability still remains within the clusters. Can this be shown overall and for individual variables?**

**R2.17.R:** Clusters explain 12% of the total variability. This information has been added to the results section. In our work, the variability cannot be explained by the variables due to the use of a MCA.

**#R2.18:** **Much emphasis in the analysis given to the fact that the phenotypes differ in mortality. But this can be expected, since the phenotypes result from clustering based on variables that are known risk-factors. So this result does not add much information to validate the usefulness of the phenotypes. Of higher interest might be analysis that compare the clusters regarding differences in clinical management or differential effects of specific treatments (compare Zhang et al 2018, Crit Care 22:347 regarding fluid therapy - this paper should be cited by the way)**

**R2.18.R:** We thank the reviewer for his/her comment and we agree with him/her. An important limitation is the lack of evaluation of heterogeneity of response to specific therapies. This data is not available in the database. This point was specified in the discussion and we added the reference proposed by the reviewer.

**#R2.19: Lines 349-366: these two paragraphs seem to be of high importance to show the possible usefulness of the obtained phenotypes, but they could be improved. I understand that previous papers discussed the possibility of differential treatment effects in subgroups stratified by baseline risk. What could the new phenotypes really add here?**

**Authors should also discuss the results regarding differential treatment effects in several trials from the Seymour et al paper (Figure 6) here. Was anything presented in the results, that could support the usefulness of the newly found phenotypes for improving trials?**

**R2.19.R:** We thank the reviewer for highlighting this point. As explained in (PMID: 26177009), RCT participants are appropriately carefully selected on the basis of their biology to be potentially responsive to the therapy under study. However, in practice, those restrictions often may not eradicate heterogeneity in baseline risk of death. We need to move beyond conventional subgroup analyses because they simply do not capture enough variation in risk. Sub-group analyses in these news phenotypes seem to be the most obvious. Actually, given realistic constraints on trial size and funding, *a priori* stratification by baseline risk seems to be more efficient to maximize the ability to detect and interpret the heterogeneity of treatment effect.

As stated in the discussion, one of the limitations of our work is related to the lack of data on a treatment. They are available in RCT like ACCESS, PROWESS and ProCESS. However, our prospective cohort is composed of unselected patients who are more representative of the general population admitted in ICU than RCT. To overcome this limitation, we have proposed the binary tree, which can be used to find our clusters prospectively, and if necessary, to apply it to the results of a negative RCT.

**#R2.20: Some language editing by a native speaker might be necessary**

**R2.20.R:** We thank the reviewer for highlighting this point; the full manuscript was reviewed in depth and the wording revised as appropriate.
