# Supplementary material for: Clinical and biological clusters of sepsis patients using hierarchical clustering
Source: PLoS One. 2021 Aug 4;16(8):e0252793. doi: 10.1371/journal.pone.0252793 (PMC8336799; doi:10.1371/journal.pone.0252793)
Supplement: Attachment — Submitted filename: Response to Editor.docx [file pone.0252793.s021.docx]

**ACADEMIC EDITOR:**

**Dear authors,**

**Thanks for your answers.**

**#AE1: The aim of the study is clearly to identify clusters of patients based on characteristic available at patients’ admission. My concerns is about clinical involvements. If your aim is to propose a different clinical strategy, you should better clarify this point.**

R.AE1: We agree, the aim of the study is clearly to identify clusters of patients based on characteristic available at patients’ admission. This sentence was added in the abstract (line 39) as follows: “The aim of the study is to identify clusters of patients based on clinical and biological characteristic available at patients’ admission.” and in the methods section (line 99) as follows: “The primary objective of our study was to identify clusters among patients with sepsis by considering data available at admission including: underlying disorders, source of infection, micro-organism, biological host response and organ failures.”.

**#AE2: It is clear that different clusters have different clinical outcomes, health care consumption, duration of stay and mortality; anyway, it is not clear why an ICU physician should use this classification to plan clinical strategy.**

R.AE2: To clarify the interest of the clustering of sepsis patient’s the sentence of the introduction section (line 77) was reformulated as follows: “As explained by J. Castela Forte, to personalize and improve treatments of sepsis, patients must be clustered into common phenotypes based on clinically objective parameters reflecting disease mechanisms. After external validation step, these clusters could be grouped by underlying causal mechanisms and will improve patient characterization; optimized design and powering of randomized control trials. Finally, these clusters can allow identifying differential response patterns by considering baseline characteristics of sepsis patients.”

**#AE3: Thanks for the sentence you added to line 358, but I would specify that further studies are necessary to better explain this clustering.**

R.AE3: We agree, sentence was modified as follow : ”First, these phenotypes will reduce heterogeneity prior to randomization; however, because they will not necessarily create better trials, further studies are necessary to better explain this clustering”.
